# Combination of metagenomic next-generation sequencing and conventional tests unraveled pathogen profiles in infected patients undergoing allogeneic hematopoietic stem cell transplantation in Jilin Province of China

**DOI:** 10.3389/fcimb.2024.1378112

**Published:** 2024-03-19

**Authors:** Hongyan Zou, Sujun Gao, Xiaoliang Liu, Yong Liu, Yunping Xiao, Ao Li, Yanfang Jiang

**Affiliations:** ^1^ Key Laboratory of Organ Regeneration & Transplantation of the Ministry of Education, Genetic Diagnosis Center, The First Hospital of Jilin University, Changchun, China; ^2^ Department of Hematology, The First Hospital of Jilin University, Changchun, China

**Keywords:** allogeneic hematopoietic stem cell transplantation, pathogen profiles, MNGs, total coincidence rate, Jilin Province

## Abstract

**Background:**

Infection is the main cause of death for patients after allogeneic hematopoietic stem cell transplantation (HSCT). However, pathogen profiles still have not been reported in detail due to their heterogeneity caused by geographic region.

**Objective:**

To evaluate the performance of metagenomic next-generation sequencing (mNGS) and summarize regional pathogen profiles of infected patients after HSCT.

**Methods:**

From February 2021 to August 2022, 64 patients, admitted to the Department of Hematology of The First Hospital of Jilin University for HSCT and diagnosed as suspected infections, were retrospectively enrolled.

**Results:**

A total of 38 patients were diagnosed as having infections, including bloodstream (*n* =17), pulmonary (*n* =16), central nervous system (CNS) (*n* =4), and chest (*n* =1) infections. Human betaherpesvirus 5 (CMV) was the most common pathogen in both bloodstream (*n* =10) and pulmonary (*n* =8) infections, while CNS (*n* =2) and chest (*n* =1) infections were mainly caused by Human gammaherpesvirus 4 (EBV). For bloodstream infection, *Mycobacterium tuberculosis* complex (*n* =3), *Staphylococcus epidermidis* (*n* =1), and *Candida tropicalis* (*n* =1) were also diagnosed as causative pathogens. Furthermore, mNGS combined with conventional tests can identify more causative pathogens with high sensitivity of 82.9% (95% CI 70.4-95.3%), and the total coincidence rate can reach up to 76.7% (95% CI 64.1-89.4%).

**Conclusions:**

Our findings emphasized the importance of mNGS in diagnosing, managing, and ruling out infections, and an era of more rapid, independent, and impartial diagnosis of infections after HSCT can be expected.

## Introduction

Hematopoietic stem cell transplantation (HSCT) is a potential radical treatment for hematological and lymphatic malignancies (Garcia et al., 2013). The overall survival rate of HSCT has been significantly improved due to the improvement of patient care after transplantation (Li et al., 2021). Of all the organ-specific complications that can occur after HSCT, incidence rate of pneumonia, which is complex and difficult to treat, can reach up to 30-60% in HSCT recipients, followed by bloodstream (Zanella et al., 2021) and central nervous system (CNS) infections. Pneumonia remains the leading cause of non-recurrent mortality after transplantation. Accordingly, not only timely but accurate diagnosis are needed to improve the prognoses and decrease the mortality.

For infection diagnosis, ideal samples include blood, cerebrospinal fluid (CSF), and bronchoalveolar lavage fluid (BALF) (Panackal, 2021; Shane et al., 2017; Berhane et al., 2021; Herbozo et al., 2021; Dorresteijn et al., 2020), while more than 60% of pathogens cannot be detected by blood, CSF, or BALF culture (Chen et al., 2020; Zhang et al., 2020; Saha et al., 2018). However, some pathogens with negative blood culture, such as *Streptococcus pneumoniae*, may result in high mortality rates (Kumar et al., 2021). Limited number of pathogens could be detected in a single experiment using hypothesis-based PCR (Marcilla-Vazquez et al., 2018) or antibody methods (Ge et al., 2021). These disadvantages hindered extensive application of conventional tests in accurate diagnosis, not to mention HSCT patients with co-infections (Jiang et al., 2021). With the first successful application of metagenomics next-generation sequencing (mNGS) in clinical infection of CNS (Wilson et al., 2014), this unbiased technology has been extensively used for diagnosing various infections (Wu et al., 2020; Chen et al., 2021; Gu et al., 2021), including co-infections (Jiang et al., 2021).

Although more and more studies used mNGS for pathogen detection in patients after HSCT, pathogen profiles still have not been reported in detail due to their heterogeneity caused by geographic region. To obtain regional pathogen profiles for guiding clinical diagnosis and treatment, we retrospectively enrolled the infected patients after HSCT and summarized the detection results revealed by mNGS and conventional tests (CT). Besides, we also evaluated the performance of mNGS and CT against final clinical diagnosis, including sensitivity, specificity, and total coincidence rate (TCR).

## Methods

### Ethics statement

This study was reviewed and approved by the Ethical Review Committee of the First hospital of Jilin University (approval no. 2022-566). All procedures followed were in strict compliance with the Ethical Review of Biomedical Research Involving Human Subjects (2016), the Declaration of Helsinki, and the International Ethical Guidelines for Biomedical Research Involving Human Subjects.

### Study population

**Figure 1:**
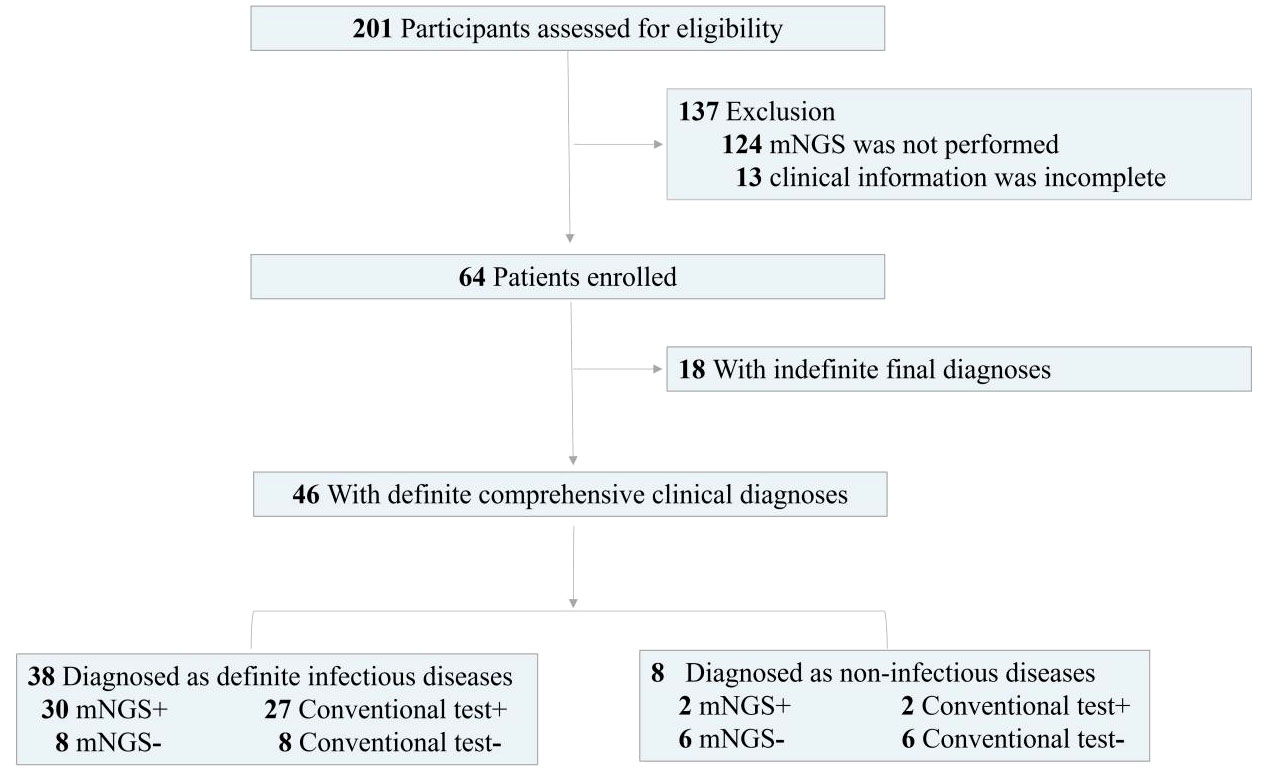
Flow diagram.

A total of 64 patients undergoing HSCT in the First hospital of Jilin University from February 2021 to August 2022 diagnosed as suspected infection were enrolled in the retrospective study (**Figure 1**). The inclusion criteria were as follows. The patients with suspected bloodstream infection were enrolled in reference to the diagnostic criteria of sepsis. Suspected CNS infection was based on acute fever (> 38.5°C) and one of the following signs: a) meningeal irritation, b) increased anterior fontanelle tension, c) stiff neck, and d) consciousness disorders. Suspected pulmonary infection was considered if the patient had new opacity on imaging examination and at least one of the following symptoms: a) respiratory distress, b) fever, c) cough, and d) peripheral leukocytosis (> 10×10^9^/L) or leucopenia (< 4×10^9^/L). The exclusion criteria included 1) mNGS was not performed and 2) clinical information was incomplete. Physical information and clinical characterization were collected. After signing informed consents, CSF, blood, and BALF samples were respectively collected from patients with suspected CNS, bloodstream, and pulmonary infections, respectively. CT included routine bacterial and fungal smears and cultures, serum antigen (Galactomannan) and antibody (Human betaherpesvirus 5 (CMV) and Human gammaherpesvirus 4 (EBV)) tests, computerized tomography, and PCR. mNGS was performed on all of samples.

### CT assay

#### Smear and culture

Gram staining and KOH test were used for bacterial and fungal smear, respectively. The bacterial and fungal cultivation were performed in the incubators at 35°C and 28°C, respectively. Culture media for bacterial culture included blood agar, chocolate agar and MacConkey agar. Besides, Lowenstein Jensen Medium was used for suspected infection of *Mycobacterium tuberculosis*. Sabauraud agar and CHROMagar Candida medium were used for fungal culture.

#### Quantitative PCR

After total DNA extraction, CMV and EBV detection were performed using commercial PCR methods (DA0121 and DA0151, DaAn Gene Co., Ltd. of Sun Yat-sen University, Guangzhou, China) according to the manufacturer’s instructions.

#### mNGS pipeline

Conventional mNGS and hybridization capture-based targeted mNGS were used to detect pathogens. The 0.6 mL blood, 0.6 mL CSF, and 1.0 mL BALF samples were respectively used to extract DNA using TIANamp Micro DNA Kit DP316 (TIANGEN Biotech, Beijing, China). Qubit 4.0 (Thermo Fisher Scientific, MA, USA) was used to measure extracted DNA concentrations. QIAseq Ultralow Input Library Kit (QIAGEN, Hilden, Germany) was used to construct metagenomics libraries (Ji et al., 2020). While for targeted NGS, the constructed library from each sample was used for hybrid capture-based enrichment of microbial probes. Inspected and qualified library was sequenced on Nextseq 550 platform (Illumina, San Diego, USA).

To remove adapter and low-quality, low complexity, and short reads of < 35 bp, raw data generated by the sequencing were filtered (Chen et al., 2021). To exclude human DNA sequences, reads were mapped to the human reference genome hg38 using bowtie2 (Langmead and Salzberg, 2012) to obtain clean reads. And then, the clean reads were blasted against a microbial genome database constructed according to the published microbial genome databases, including reference sequence database at National Center for Biotechnology Information. Finally, microbial information at species level can be obtained.

As control, using the same procedure and bioinformatics analysis, negative and positive controls were also set during the mNGS tests of samples from patients. The reads per million (RPM) of each detected pathogen were calculated. For the detected bacteria (*Mycobacterium* excluded), fungi (*Cryptococcus* excluded), or parasites, a positive mNGS result was defined when the microorganism was not detected in the negative control (‘No template’ control, NTC) and genome coverage of detected sequences belonged to this microorganism ranked top10 among the microbes in the same genus or when its ratio of RPM_sample_ to RPM_NTC_ was (RPM_sample_/RPM_NTC_) > 10 if the RPM_NTC_≠0. For viruses, *Mycobacterium*, and *Cryptococcus*, a positive mNGS result was considered when the virus was not detected in NTC and at least 1 specific read was mapped to species or when RPM_sample_/RPM_NTC_ was > 5 if the RPM_NTC_≠0. The interpretation of mNGS results was performed by 2-3 clinical adjudicators. Positive mNGS results were defined according to whether the pathogens were the most commonly reported pathogens or the infections by the pathogens were in accordance with clinical features of patients (Zhang et al., 2020; Zhang et al., 2023).

### Diagnostic assessment

According to mNGS results (including both positive and negative mNGS results), complete laboratory examinations, the treatment response of the patients, and clinical experiences, the final clinical diagnoses and causative pathogens were independently made by 2-3 clinicians with expertise in infectious diseases. According to final comprehensive clinical diagnoses, we divided enrolled patients into definite and indefinite clinical diagnosis groups. Definite clinical diagnosis group included patients with infectious and non-infectious diagnoses. Indefinite clinical diagnosis group included patients whose clinical characteristics or laboratory examinations were not adequate for diagnoses and patients lost during follow-up duration.

Given semi-quantitative characteristics of mNGS, dynamic surveillance of infections can be conducted (Zhang et al., 2020). To evaluate the efficacy of medication in reference to mNGS results, comparison was performed as follows. Firstly, some microbes detected by the 1^st^ mNGS were diagnosed as causative pathogens of patient. Subsequently, if specific reads of the pathogen detected by the 2^nd^ mNGS decreased after effective anti-microbial treatment in reference to mNGS results and the infection symptoms were partially improved, we defined that mNGS can provide positive reference for treatment strategies. Otherwise, if continuous two or more mNGS results were negative at short interval and patient was recovered without anti-microbial treatment, we defined that negative mNGS results can be used to rule out infection.

### Statistical analysis

Counts and percentages were presented for independent variables. Mean ± standard error (SE) was calculated for continuous variables with normal distributions, while medians and interquartile ranges (IQRs) were used for abnormal distributions. Confidence intervals were calculated according to the formula: CI=Average ± 1.96SE. Spearman correlation analysis was conducted, and *P* value of < 0.05 were considered statistically significant. The data were analyzed using IBM SPSS 25.0 and R 4.1.1.

### Data availability

Sequencing data were deposited to the National Genomics Data Center under accession numbers PRJCA014245 and CRA009447. The authors declare that the main data supporting the findings are available within this article. The other data generated and analyzed for this study are available from the corresponding author upon reasonable request.

## Results

### Clinical characteristics of the patients undergoing HSCT

**Figure 2:**
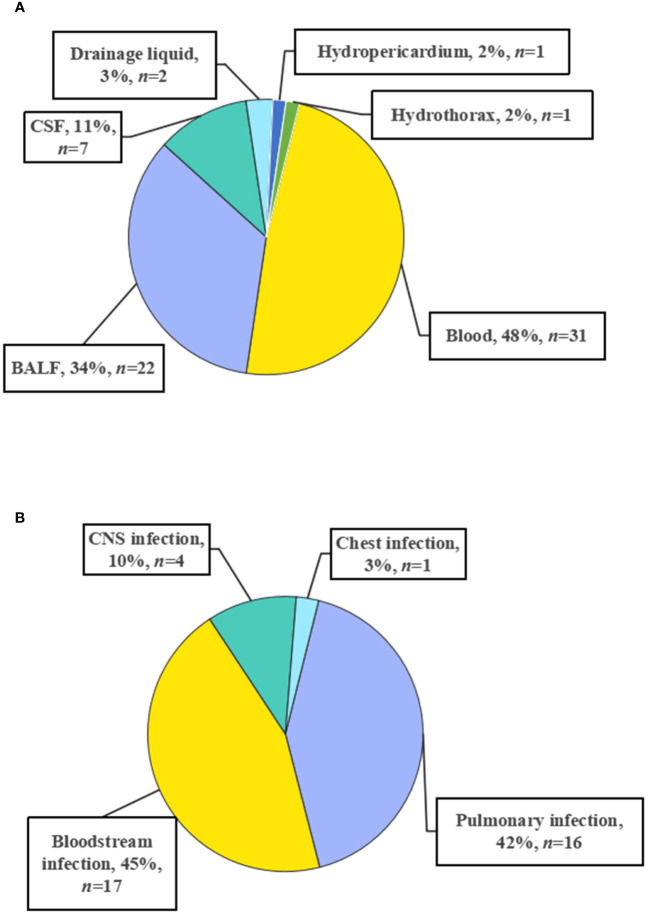
Sample type **(A)** and infection site **(B)**.

A total of 64 patients undergoing HSCT diagnosed as having suspected infections were enrolled in the retrospective study (**Figure 1**), including 33 males and 31 females. The patients underwent transplantation due to different kinds of leukemias, including acute myeloid leukemia (*n* =20), acute lymphoblastic leukemia (*n* = 14), acute leukemia (myeloid + lymphoblastic leukemia) (*n* = 1), chronic myeloid leukemia (*n* = 1), myelodysplastic syndrome (*n* = 9), aplastic anemia (*n* = 1), and lymphoma (*n* = 2) (**Table 1**). For further diagnoses, samples were collected from 64 patients for mNGS and conventional tests, including 31 blood, 22 BALF, 7 CSF, 2 drainage liquid, 1 hydropericardium, and 1 hydrothorax samples (**Figure 2A**). According to final comprehensive clinical diagnoses, we divided enrolled patients into definite (*n* = 46) and indefinite (*n* = 18) clinical diagnosis groups. Among the definite group, a total of 38 patients were diagnosed as having infections, including bloodstream (*n* = 17), pulmonary (*n* = 16), CNS (Viral encephalitis, *n* = 4), and chest (*n* = 1) infections (**Figure 2B**).

**Table 1:** Baseline characteristics of patients after HSCT.

	Cases (*n* = 64)	
Sex (*n*, %)	Male	33 (51.6%)
	Female	31 (48.4%)
Age (years)	Median (min,max)	40.4 (0.9, 75)
WBC (×10^9^/L)	Average	3.4 ± 3.2
PCT (ng/mL)	Average	7.9 ± 26.7
CRP (mg/L)	Average	69.9 ± 83.1
Underlying disease (*n*, %)	Total	6 (9.4%)
	Hypertension	2 (3.1%)
	Post-operation of lung cancer	1 (1.6%)
	Hepatitis B	1 (1.6%)
	Food allergy	1 (1.6%)
	Cholecystectomy	1 (1.6%)
Clinical diagnosis (n, %)	Aplastic anemia	1 (1.6%)
	Acute lymphoblastic leukemia	14 (21.9%)
	Acute myeloid leukemia	20 (31.3%)
	Myelodysplastic syndrome	9 (14.1%)
	Chronic myeloid leukemia	1 (1.6%)
	Lymphoma	2 (3.1%)

### Regional pathogen profiles in different systems

**Figure 3:**
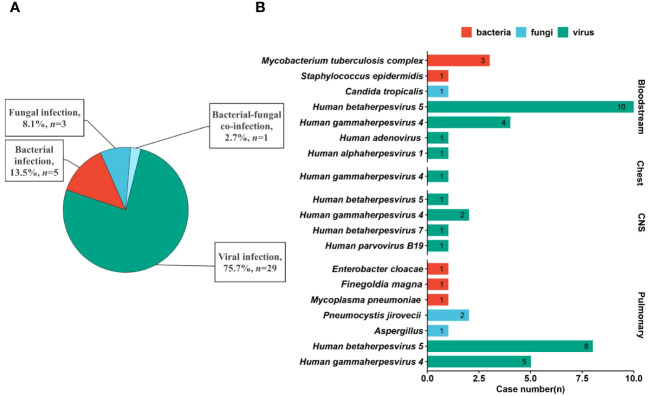
Causative pathogens in different systems. **(A)** Infection type in patients after HSCT. **(B)** Pathogen profiles causing bloodstream, chest, CNS, and pulmonary infections based on final clinical diagnoses.

There are quite differences in pathogen profiles among different systems (**Figure 3**). More than 75% of infections were caused by viruses and only 1 patient had bacterial-fungal co-infection. Bacteria, fungi, and viruses were diagnosed as causative pathogens in bloodstream and pulmonary infections, while pathogens causing CNS and chest infections were found to be viruses. For viruses, CMV was the most common pathogen in both bloodstream (*n* = 10, detection rate of 58.8%) and pulmonary (*n* = 8) infections, while CNS (*n* = 2) and chest (*n* = 1) infections were mainly caused by EBV. Besides, EBV can also cause bloodstream (*n* = 4) and pulmonary (*n* = 5) infections with high detection rates.

Apart from viruses, *Pneumocystis jirovecii* (*n* =2), *Aspergillus* (*n* =1), *Enterobacter cloacae* (*n* =1), *Finegoldia magna* (*n* =1), and *Mycoplasma pneumoniae* (*n* =1) were detected in pulmonary infection. For bloodstream infection, *Mycobacterium tuberculosis* complex (*n* =3), *Staphylococcus epidermidis* (*n* =1), and *Candida tropicalis* (*n* =1) were diagnosed as causative pathogens. Identification of regional pathogen profiles can help guide clinical empirical treatment before accurate diagnoses to improve prognosis of patients.

### Comparison between mNGS and CT

**Figure 4:**
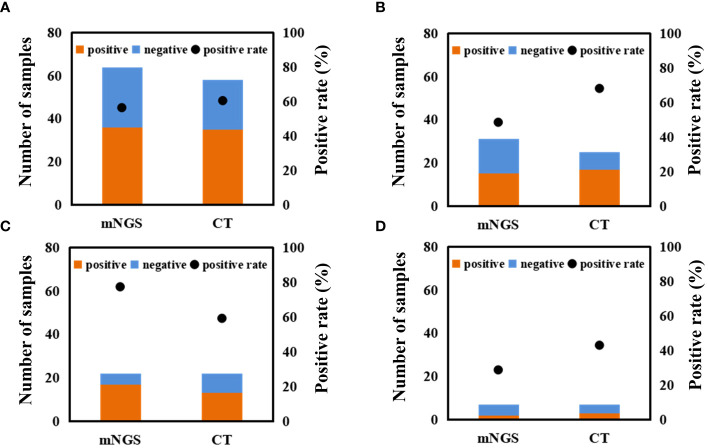
Comparison between mNGS and CT in microbial detection using different samples. Differences in microbial detection between mNGS and CT using different samples. **(A)** all of samples. **(B)** blood sample. **(C)** BALF sample. **(D)** CSF sample. ‘CT’ represents conventional tests.

Differences in positive rate between mNGS and CT for different samples were found (**Figure 4** and **Supplementary Table S1**). For BALF sample, the positive rate of mNGS (77%) was significantly higher than that of CT (59.1%). For blood sample, mNGS can detect microbes from 15 out of 31 samples (48.4%), while CT can detect microbes from 17 out of 25 samples (68.0%). Besides, positive rates of both mNGS and CT for CSF sample were low. Overall, the positive rate of mNGS (56.3%) was close to that of CT (60.3%). Accordingly, we proposed that mNGS might be an alternative or supplemental examination for diagnosing infections when CT fails.

**Figure 5:**
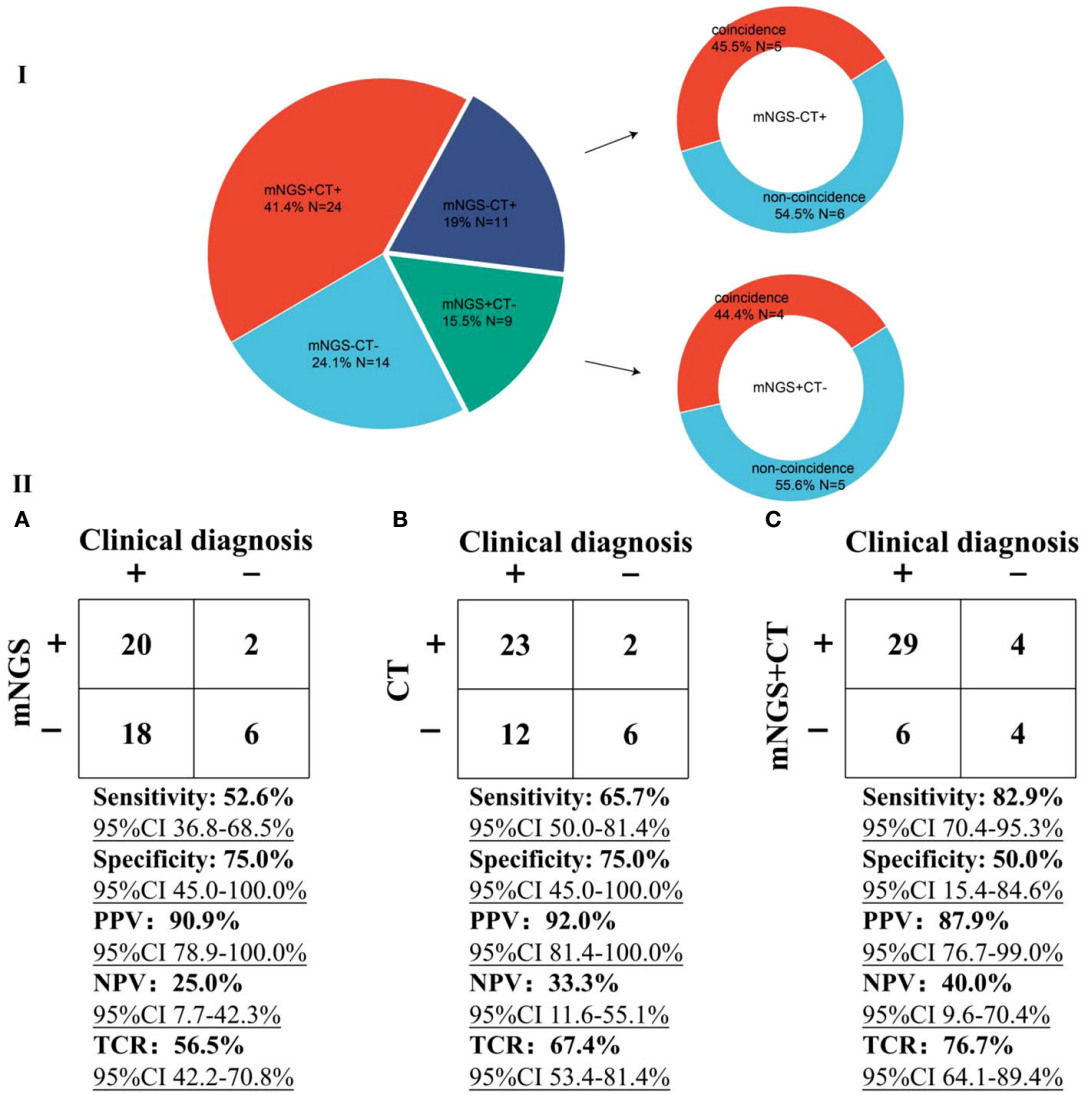
Performance comparison between mNGS and CT. I, Comparison of differences in detection between mNGS and CT. ‘N’ was the number of cases. ‘+’ and ‘-’ represent positive and negative results, respectively. II, Performance of mNGS **(A)**, CT **(B)**, and mNGS combined with CT **(C)** against final clinical diagnoses.

In our study, mNGS was performed on the whole patients enrolled, while CT was performed on 58 patients (**Figure 5**). There were 24 patients with both mNGS and CT positive, and 14 patients with both mNGS and CT negative, while inconsistent results between mNGS and CT were found in more than 34% of 58 patients. For the patients with mNGS positive and CT negative (*n* =9), about half of patients (4 out of 9) can be directly diagnosed only by mNGS results with positive coincidence rate of 44.4% against final clinical diagnoses. A same trend was found in the patients with mNGS negative and CT positive. The above results indicate that there were no significant differences in detecting microbes between mNGS and CT.

Taking final clinical diagnoses as gold standard, we further compared the performance of mNGS and CT in diagnosing infections (**Figure 5**). The sensitivity of mNGS was slightly lower [52.6% (95% confidence interval (CI) 36.8-68.5%)] than that of CT [65.7% (95% CI 50.0-81.4%)]. The specificities of both mNGS and CT were 75%. Positive predictive values (PPV) of both mNGS [90.9% (95% CI 78.9-100.0%)] and CT [92.0% (95% CI 81.4-100.0%)] were >90%, while negative predictive values (NPV) of both mNGS (25.0% [95% CI 7.7-42.3%)] and CT [33.3% (95% CI 11.6-55.1%)] were low. Besides, more than half of mNGS (TCR, [56.5% (95% CI 42.2-70.8%)] and CT [67.4% (95% CI 53.4-81.4%)] results were consistent with the final clinical diagnoses. Most importantly, compared with mNGS or CT, mNGS combined with CT can identify more causative pathogens with higher sensitivity of 82.9% (95% CI 70.4-95.3%), and the TCR can reach up to 76.7% (95% CI 64.1-89.4%). The above results unravel that mNGS can be considered as a supplement to CT for infection diagnosis in patients after HSCT mainly infected by viruses.

### Semi-quantitative value of mNGS

**Figure 6:**
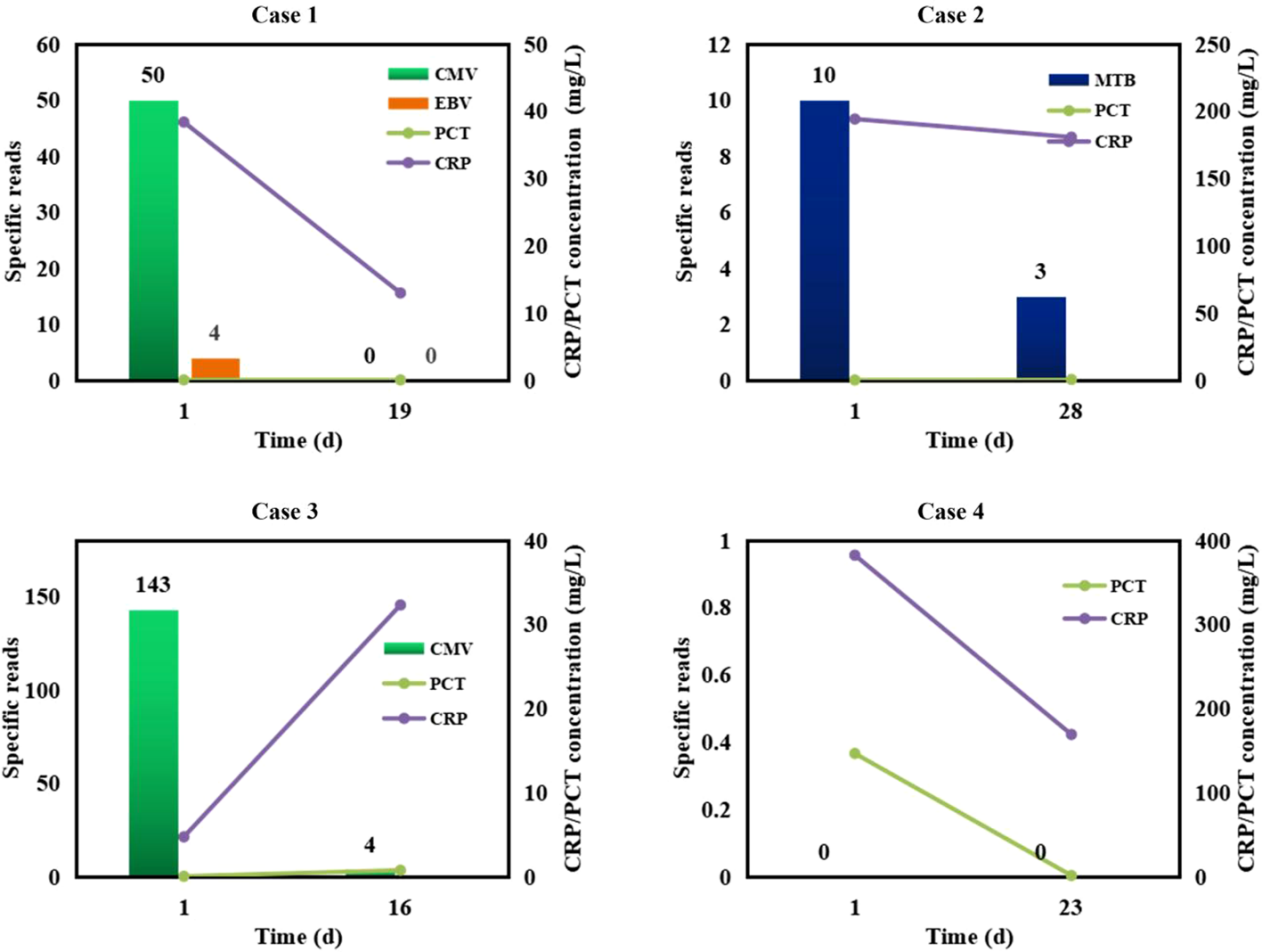
Semi-quantitative value of mNGS in the dynamic surveillance of infections. Case 1, CMV and EBV infections. Case 2, *M.tuberculosis* (MTB) infection. Case 3, CMV infection. Case 4, non-infection.

Given semi-quantitative characteristics of mNGS, we performed mNGS on some patients at different intervals to further verify the efficacy of medication in reference to mNGS results (**Figure 6**). In cases 1-3, CMV, EBV, or *M.tuberculosis* were detected by the 1^st^ mNGS and diagnosed as causative pathogens, respectively. After effective anti-microbial treatment, specific reads detected by the 2^nd^ mNGS decreased to low level or even to 0, and the infection symptoms of these cases were partially improved. We also use mNGS to rule out infection in some case. Case 4 was finally diagnosed as non-infection disease by the two negative mNGS results at 28 d interval. The above results further emphasize the importance of mNGS in providing reference for treatment strategies.

## Discussion

To the best of our knowledge, this is the first report on summarizing regional pathogen profiles of infected patients after HSCT in Jilin Province of China revealed by mNGS and CT. Bloodstream infection was the most common infection in patients after HSCT, followed by pulmonary infection, CNS infection, and chest infection. Taking final clinical diagnoses as gold standard, we found that more than 75% of infections were caused by viruses, and CMV and EBV were found to be the most common pathogens. Besides, mNGS can be considered as a supplement to CT for infection diagnosis in patients after HSCT infected mainly by viruses.

We found that the performance of mNGS in diagnosing infection was not better than that of CT, which was contrary to the results of previous studies (Qu et al., 2022; Gu et al., 2021; Chen et al., 2020; Yu et al., 2022). It was reported that the sensitivity of mNGS was determined by the pathogen DNA ratio in sample (Ebinger et al., 2021). Host depletion methods, such as differential lysis method, can filter human DNA (Ji et al., 2020), increasing pathogen DNA ratio (Ji et al., 2020; Gu et al., 2021; Thoendel et al., 2018) at the expense of some viruses, parasites, and bacteria (Ji et al., 2020). Besides, host depletion methods may bring in contamination of engineered strains from reagents (Gu et al., 2021), decreasing the detection accuracy (Han et al., 2021). In our study, viral infection accounted for more than 75%, and host depletion method was also included in mNGS pipeline. However, there was no need to deplete host DNA for PCR in CT used in our study, avoiding the loss of viral genes. The above might be the reasons why the performance of mNGS was slightly lower than that of CT. Furthermore, we found that mNGS combined with CT can identify more causative pathogens and the TCR can reach up to 76.7%. Accordingly, we propose that mNGS and CT should be simultaneously performed on patients mainly infected by viruses to improve diagnostic accuracy.

In our study, CMV and EBV were found to be the most common pathogens causing infections in patients after HSCT. EBV is responsible for 2-5% of viral encephalitis and meningitis patients, and a review covering a 10-year period revealed that EBV was occasionally detected in CSF using PCR (Lee et al., 2021). Latent infections in B lymphocytes and myeloid cells are essential for persistence and transmission of EBV and CMV (Fragkou et al., 2021), respectively, and EBV is estimated to infect >90% of adults worldwide (Hong et al., 2021). Besides, treatment-related immunosuppression provides conditions for reactivation and infection of latent viruses (Fragkou et al., 2021; Arruti et al., 2017), and viral reactivation in patients after HSCT is associated with significant morbidity and mortality (Fragkou et al., 2021). Previous studies also found that EBV was the leading cause of CNS viral infection in patients after HSCT (Qu et al., 2022; Liu et al., 2019), which was consistent with our study (**Figure 3**). Meanwhile, CMV was found to be one of the most significant pathogens causing morbidity and mortality in patients after HSCT (Imlay and Kaul, 2021; Haidar et al., 2020), and we also found that the most common pathogen was CMV in patients after HSCT. Given the high risk of viral reactivation, we propose that clinicians should strictly perform seropositivity screening for viruses on both donors and candidates according to pre-transplantation evaluation guidelines (Fragkou et al., 2021; Tomblyn et al., 2009; U.S. Food and Drug Administration, 2019), to judge whether pre-emptive treatment should be conducted.

Furthermore, reads of semi-quantitative mNGS can reflect disease progression and treatment efficacy (Ge et al., 2021; Zhang et al., 2020; Ai et al., 2018). Besides, the value of mNGS as being a “rule-out” role (O'Grady, 2019; Chiu and Miller, 2019) is beneficial to minimizing the abuse of anti-microbial drugs (Ge et al., 2021). Zhang et al. performed several mNGS tests at different intervals on nine patients to evaluate the role of mNGS during the treatments of CNS infections, and found that for the patients with effective antimicrobial treatment in reference to mNGS results, mNGS semi-quantitative sequencing reads correlated with CSF WBC and glucose ratio level (Zhang et al., 2020). Chen et al. also exhibited the important role of repeated mNGS tests in diagnosing and managing *Escherichia coli* infection of neonates (Chen et al., 2022). The findings of our study provide more evidences to further emphasize the importance of mNGS in diagnosing, managing, and ruling out infections, and an era of more rapid, independent, and impartial diagnosis of infections in patients after HSCT can be expected. Given the high cost of mNGS, it needs skilled staff to be correctly performed.

## Limitations

RNA mNGS was not performed on the patients in this study, and more samples from multiple hospitals are needed to further evaluate the performance of both DNA and RNA mNGS in diagnosing infections in patients after HSCT. More negative controls from non-infectious cases should be included to further detect the potential of mNGS in ruling out infection. In addition, the potential of mNGS to help with the timely adjustment of treatments should also be evaluated.

## Conclusions

Our findings highlight the importance of mNGS combined with CT in diagnosing infections of patients after HSCT, especially for viral infection. Bloodstream infection was the most common infection in patients after HSCT, followed by pulmonary infection, CNS infection, and chest infection. Most of infections were caused by viruses, including CMV and EBV. Based on our findings, extensive application of mNGS in diagnosing infections after HSCT could be expected.

## Data availability statement

The datasets presented in this study can be found in online repositories. The names of the repository/repositories and accession number(s) can be found below: https://ngdc.cncb.ac.cn/search/?dbId=bioproject&q=PRJCA014245, PRJCA014245.

## Ethics statement

This study was reviewed and approved by the Ethical Review Committee of the First hospital of Jilin University (approval no. 2022-566).

## Author contributions

HZ: Writing – original draft, Writing – review & editing. SG: Writing – original draft, Writing – review & editing. XL: Writing – original draft. YL: Formal analysis, Writing – original draft. YX: Writing – original draft. AL: Writing – original draft. YJ: Funding acquisition, Writing – original draft, Writing – review & editing.
